# Spatio-temporal stratified associations between urban human activities and crime patterns: a case study in San Francisco around the COVID-19 stay-at-home mandate

**DOI:** 10.1007/s43762-022-00041-2

**Published:** 2022-06-06

**Authors:** Tongxin Chen, Kate Bowers, Di Zhu, Xiaowei Gao, Tao Cheng

**Affiliations:** 1 SpaceTimeLab for Big Data Analytics, Department of Civil, Environmental and Geomatic Engineering, University College London, Gower Street, London, WC1E 6BT UK; 2 Department of Security and Crime Science, University College London, Tavistock Square, London, WC1H 9EZ UK; 3 Department of Geography, Environment and Society, University of Minnesota, Twin Cities, 55455 Minneapolis US

**Keywords:** Spatio-temporal stratified association, Crime pattern analysis, Human activity, Social sensing, COVID-19

## Abstract

Crime changes have been reported as a result of human routine activity shifting due to containment policies, such as stay-at-home (SAH) mandates during the COVID-19 pandemic. However, the way in which the manifestation of crime in both space and time is affected by dynamic human activities has not been explored in depth in empirical studies. Here, we aim to quantitatively measure the spatio-temporal stratified associations between crime patterns and human activities in the context of an unstable period of the ever-changing socio-demographic backcloth. We propose an analytical framework to detect the stratified associations between dynamic human activities and crimes in urban areas. In a case study of San Francisco, United States, we first identify human activity zones (HAZs) based on the similarity of daily footfall signatures on census block groups (CBGs). Then, we examine the spatial associations between crime spatial distributions at the CBG-level and the HAZs using spatial stratified heterogeneity statistical measurements. Thirdly, we use different temporal observation scales around the effective date of the SAH mandate during the COVID-19 pandemic to investigate the dynamic nature of the associations. The results reveal that the spatial patterns of most crime types are statistically significantly associated with that of human activities zones. Property crime exhibits a higher stratified association than violent crime across all temporal scales. Further, the strongest association is obtained with the eight-week time span centred around the SAH order. These findings not only enhance our understanding of the relationships between urban crime and human activities, but also offer insights into that tailored crime intervention strategies need to consider human activity variables.

## Introduction

Crimes do not occur randomly in time or space (Brantingham et al. 1981, 2017). The disparity of crime events distributions across places, streets and communities has been confirmed by amounts of research hitherto (Shaw and McKay 1942; Sherman et al. 1989; Sampson and Groves 1989; Weisburd 2015). *Crime pattern theory* suggests that the variation of urban crime distribution are shaped by heterogeneous urban environmental characterizations promoting the various possibility of crime opportunities. Specifically, urban areas that are temporally concentrated by the intersections of routine activities between potential offenders and suitable victims/targets would experience more crime occurrences, while areas with high-presence of guardians are found to have low-level crime events (Clarke 1995; Cohen and Felson 1979; Brantingham et al. 2017; Wortley and Townsley 2016).

Past studies have evaluated amounts of urban population characterizations with unevenly crime distributions to disentangle the mechanism of crime pattern. A common observation is that the heterogeneous nature of populations promote a core antecedent to crime across different urban areas and time spans, such as demographic features, social inequalities (Sampson et al. 1997; Sampson 2019; Sampson and Raudenbush 1999; Rukus and Warner 2013; Murray et al. 2012). Previous research has found the heterogeneity of land-use functions as a proxy of human activity in urban areas (Song et al. 2017; Stucky and Ottensmann 2009; Kinney et al. 2008; Boivin 2018). This representation is underpinned by the association between urban crime characteristics and the ambient population which is likely to contain potential victims, offenders and also crime preventers (Andresen 2011; Rengert and Wasilchick 1985).

Spatial heterogeneity as the association between variables distributing heterogeneously in space is generally acknowledged in urban crime studies (Cahill and Mulligan 2007; Graif and Sampson 2009). Unlike the disadvantage of the global perception that considers the relationships between observation variables across geospatial units in a constant way, spatial heterogeneity investigating and portraying the associations between crime patterns and risk factors can help to unveil the complex crime generation mechanism (Chen et al. 2020; Boivin 2018). Correspondingly, leveraging the concept of strata, *spatial stratified heterogeneity* (SSH) as a type of quantifying the spatial heterogeneity, measures the association between dependent and explanatory variables by the variances in the stratified observations across geospatial strata (Wang et al. 2010; Wang et al. 2016; Song and Wu 2021). The use of strata is ubiquitous in geospatial data types, such as urban function zones, climate zones, geographic divisions (Wang et al. 2016, 2010, Wang and Hu 2012). Due to advantage of explanatory variable examination, the SSH has been widely examined in associations across various variables regarding various geospatial strata, e.g., detecting disease risk in public health (Wang et al. 2010; Gu et al. 2019), exploring environmental problems in ecology (Su et al. 2020) and explaining the determinants of housing price in urban studies (Zhu et al. 2021; Wang et al. 2017).

In light of spatial heterogeneity phenomena, examining the associations between particular human activities and the corresponding crime patterns in space and time are essential to understanding the distributions of crime driven by various risk factors (Browning et al. 2021). Obviously, research attempting to explain crime pattern dynamics that relies on a static picture of fixed urban characterisation (e.g., land-use) strata is limited (Sohn et al. 2018), as it cannot reflect the temporal dynamic of populations across urban ares. Besides, artificial spatial stratification based on experienced knowledge may be contradicted with the strata in nature and cannot reveal the true spatial heterogeneity (Wang and Hu 2012)

In recent years, the widespread proliferation of information and communication techniques (ICTs) has fostered massive amounts of user-generated big geo-data representing the interactions between individuals and geographic environments (Goodchild 2007; MacEachren 2017). As citizens are actively sharing their digital footprints, various forms of human activity data with the associated spatio-temporal stamps are being collected (Jenkins et al. 2016). Moreover, corresponding social sensing analytics (Liu et al. 2015, Zhu et al. 2017, 2020) provide opportunities for taking large scale data-driven approaches to analyzing the socioeconomic dynamics and human movement within cities. This makes it possible to characterize the temporal human activity patterns at different geospatial scales (Liu et al. 2020). Hence, dynamic human activities within places, neighborhoods or urban areas can now be directly modelled by large-scale and high-resolution geo-data. There is now a need for more empirical research based on such spatio-temporal data to help capture the spatial and temporal association between human activities and crime patterns from a dynamic person-place interaction perspective.

Specifically, it is evident that the outbreak and spread of the COVID-19 pandemic has influenced human activities and population dynamics in global cities in an unprecedented way (Bonaccorsi et al. 2020; Kissler et al. 2020; Huang et al. 2020). Specifically, urban citizen activities have been shifted by the public order associated with pandemic containment, such as lockdowns and stay-at-home (SAH) mandates (Zhu et al. 2021). This has been accompanied by non-essential businesses, public transport and entertainment facilities closing down or restricting their activities. Soon after human activity changed as a result of these enforced polices in cities, a growing body of literature emerged addressing crime pattern changes by examining the crime variance in two significant periods (i.e., before/after SAH). Theses studies have predominately been city-level analyses using aggregated crime time series (Ashby 2020; Mohler et al. 2020). As a general rule, the COVID and crime studies have found significant reductions in property crime (Gerell et al. 2020; Campedelli et al. 2021; Felson et al. 2020), but increases in several types of violent crime, such as domestic violence (Leslie and Wilson 2020; Piquero et al. 2020). Additionally, community-level analysis of crime change during SAH suggests that stable social, economic, and demographic variables, such as income diversity, vacant housing and population size, are associated with significant crime reduction (Campedelli et al. 2020). Recent studies concerning the interpretation of crime pattern change during the pandemic have the limitation that they use non-varying factors in urban areas (e.g., census survey data) as predictors of variation in pandemic related crime trends. These obvious cannot address the spatio-temporal association between crime pattern and human activity in the context of social or economic environmental change.

Though some studies have added in population movement variability in the COVID and crime models (e.g., Google mobility) to better reflect the dynamic shifts in routine activities (Halford et al. 2020; Payne et al. 2020; Langton et al. 2021). Studies mainly focus on global time series changes at country or city level, as data is only provided at these levels of aggregation. These efforts fail to disentangle the spatial-temporal association between stratified explanatory variables (e.g., human activities) and the heterogeneous urban crime patterns. Moreover, such global analysis might neglect the nuances that are useful in informing more tailored crime prevention approaches. The common practice of splitting analytical periods into before and after the start-date of SAH does not allow consideration of temporal continuity or gradual change in either crime or human activity neglecting the alternative possibilities to a single sudden.

Herein, our intention in this paper is to explore the spatial-temporal stratified association between dynamic human activity and crime during the SAH order at different temporal observation periods. In Section 2, we proposed the analytical framework to reveal the processes of spatio-temporal stratified heterogeneity (STSH) based on human activity and crime patterns. First, we introduce a new approach to characterizing changes of human activity in CBGs at fine-granular spatial units. We propose a data-driven spatial stratification using of *functional zoning* based on the similarity of human activity patterns to identity meaningful human activity zones (HAZs) within a preset temporal observation period (TO). We then describe how the method of STSH testing can be used to identify the association between the HAZs and crime distribution across CBGs. In Section 3, we apply these methods in a case study using data from San Francisco, USA. This begins with a description of the data and the study area used in this work. The results examine the crime distributions within identified HAZs, and report spatio-temporal association matrices representing relationships between human activity and crime. Lastly, Section 4 assesses the implication of the resulting STSH associations for different crime types and Section 5 summary our findings and suggests further research avenues within this area.

## Methodology

In this section, we proposed a framework for detecting the spatio-temporal stratified heterogeneity (STSH) as the association between human activity and crime distributions. Here, we start with the clarification of several key concepts of definitions in our analysis procedures.

**Strata**: Strata is consist of a set of stratum clustered from basic geographic units according to the similarity of variable observations.

**Human activity zone** (HAZ): Human activity zone is a stratum that obtaining the homogeneity of temporal human activity patterns at several geospatial units. In our analysis, we retrieve the human activity zones by leveraging a hierarchic clustering strategy.

**Spatial stratified association** (SSA): Spatial stratified association, i.e., spatial stratified heterogeneity of explanatory variables in the response variables across geospatial units and can be utilised for describing the association between explanatory variables and response variables. In our study, explanatory variables and response variables are human activity pattern and crime pattern, respectively.

**Spatio-temporal stratified association** (STSA): Spatio-temporal stratified association, i.e., Spatio-temporal stratified heterogeneity of explanatory variables in the response variables across geospatial units in different observation periods. In our study, spatio-temporal stratified association aims to detect the dynamic associations between variables.

### Analytical framework

**Fig. 1:**
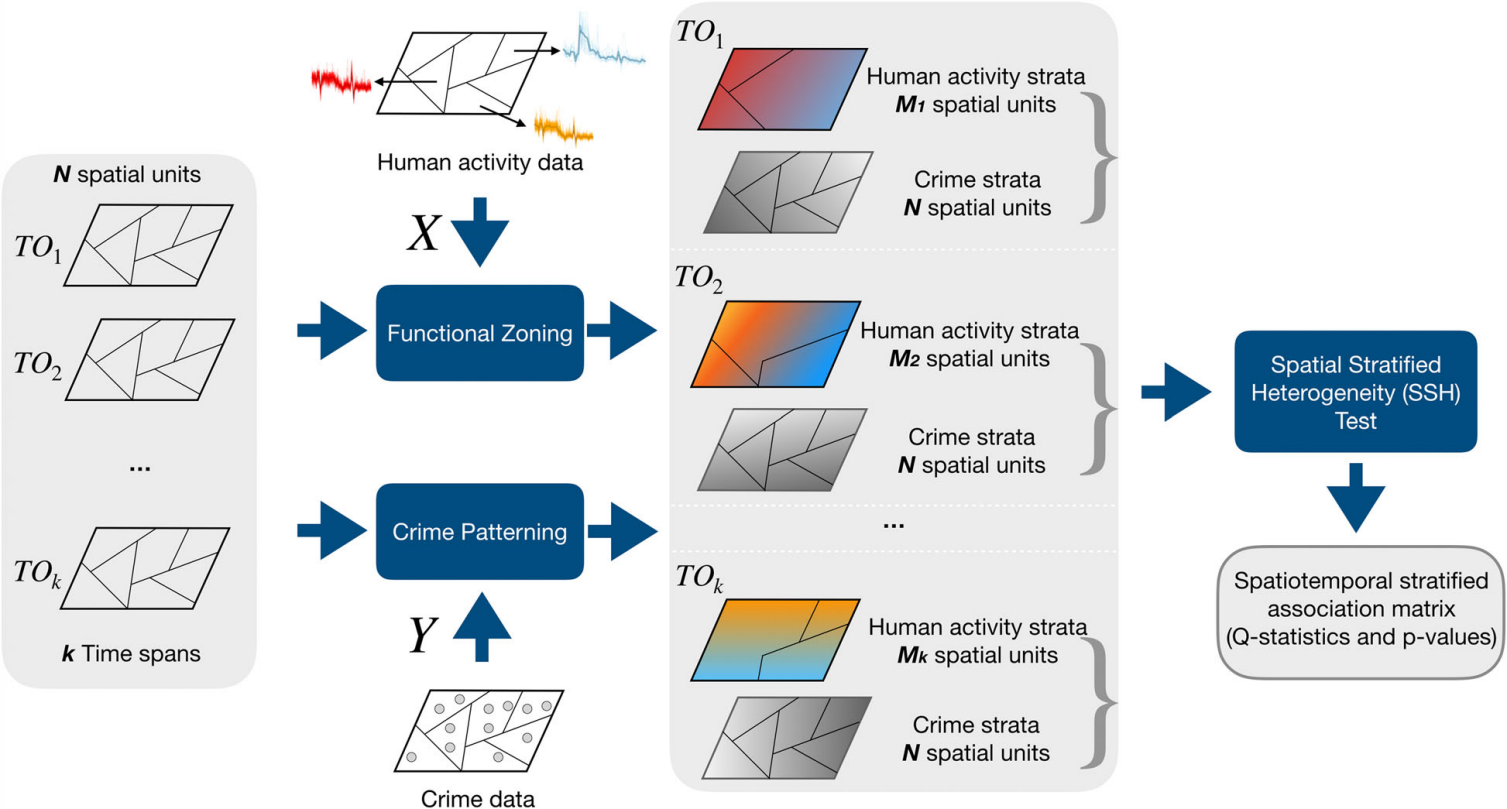
Analytical framework for detecting the spatio-temporal stratified association between human activities and crime patterns

Figure 1 indicates the main procedures and analytical framework for detecting the spatio-temporal stratified association between human activities and crime patterns in geospatial strata. The method is organized as follows. We first preset *K* time observation periods (TOs) for representing the temporal variance within an urban region with *N* geospatial units. These *K* TOs enable us to capture the human activity patterns within sequential and overlapped time duration. Then, in the functional zoning process, an agglomerative clustering algorithm is utilized to generate human activity zones (HAZs) based on the similarity of human activity patterns at different spatial units during one of the TOs, i.e., *M* HAZs are generated from *N* geospatial units (*N*≥*M*) in a given *TO*_*k*_. Crime incidents volumes are aggregated to the *N* geospatial units for each TO to quantify the crime distributions. Collectively, this generates HAZs strata with *M* units as variable *X* and crime pattern strata with *N* units as variable *Y*. These variables are then used in a STSH test which yields the spatio-temporal stratified association index (*Q* statistic) between the two strata. Repeating these analytical steps enables us to bring together and compare the association indices for a number of different crime types across *K* TOs.

### Dynamic functional zoning based on human activities

In analogy to ”the urban functional zone”, which is a kind of regional space that provides specific functions for human activities and reflects the land use type in a city (Xing and Meng 2018), we use human activity zones (HAZs) to refer to a set of spatial unit clusters, where each HAZ exhibits a certain type characterized by the routine activities of the citizens. As geographic strata, HAZs as the representation of human activity pattern are used in the STSH testing procedures and several HAZs in different time periods can support the examination of spatio-temporal stratified association.

The objective of functional zoning is, for each of the given time observation periods (a given TO), to generate *M* human activity zones based on the human activity data collected at the *N*(≥*M*) spatial administrative units. The dynamic functional zoning is carried out using the following steps:

- Step 1. Collecting spatio-temporal data that characterizes human activity patterns.
- Step 2. Slicing the data into different temporal observation periods.
- Step 3. Hierarchical clustering of human activity patterns.

We begin by collecting the spatio-temporal data at the basic spatial level with *N* units. The required data should be able to reflect temporal information about human activities, such as the number of people and the frequency of visit in each spatial unit *S*_*i*_. Then, the human activity data for *S*_*i*_ is organized as a vector *A*_*i*_: $A_{i}=[a_{i}^{0},a_{i}^{1},\dots,a_{i}^{T-1},a_{i}^{T}], i=1,2,\dots,N$, with $a_{i}^{T}$ denoting the absolute value at time *T* in *S*_*i*_.

Then, we define *k* temporal observation periods *TO*_1_,*TO*_2_,…, and *TO*_*k*_ to enable the dynamic clustering. Each period *TO*_*k*_ refers to a unique time span from *t*_*k*,0_ to *t*_*k*,*t*_, which is meaningful from a research perspective. Hence, for time span *TO*_*k*_, the corresponding absolute human activity pattern $A_{i}^{TO_{k}}$ would be $[a_{i}^{t_{k,0}},a_{i}^{t_{k,1}},\dots,a_{i}^{t_{k,t-1}},a_{i}^{t_{k,t}}]$.

In order for HAZ classification to consider both the dynamic function and capacity of spatial units regarding human activities, we followed hierarchical procedures to perform the unsupervised two-level clustering similar to Zhu et al. (2017). In detail, with respect to the dynamic function of spatial units, the first-level clustering is performed based on the standardized variation signatures of the human activity. While, the second-level clustering mainly considers the human activity capacity of spatial units, which is reflected in the absolute volumes of observed human activities.

Based upon the absolute vector $A_{i}^{TO_{k}}$, which can be viewed as the capacity of *S*_*i*_ in temporal period *T*_*k*_, we further calculate a row-standardized vector $V_{i}^{TO_{k}}$ to represent variation in activity patterns over time,

$V_{i}^{TO_{k}}=[\frac {a_{i}^{t_{k,0}}-\mu _{i}}{\sigma _{i}},\frac {a_{i}^{t_{k,1}}-\mu _{i}}{\sigma _{i}},\dots,\frac {a_{i}^{t_{k,t-1}}-\mu _{i}}{\sigma _{i}},\frac {a_{i}^{t_{k,t}}-\mu _{i}}{\sigma _{i}}]$, where $\mu _{i}=\sum _{1}^{t}\frac {a_{i}^{t_{k,t}}}{t}$ and $\sigma _{i}=\sqrt {\frac {\sum _{1}^{t}(a_{i}^{t_{k,t}}-\mu _{i})^{2}}{t-1}}$.

For each temporal observation period *TO*_*k*_, we repeatedly conduct the following hierarchical clustering to generate a functional zoning of HAZs:

(1) Use the row-standardized vector $V_{i}^{TO_{k}}$ to execute the agglomerative clustering algorithm^1^, generating *m*_1_ first-level types that base on the similarity of pattern variations. Here, we use the silhouette coefficient as the measurement index of similarity (this index ranges from 1 to +1, where a high value denotes that the vector is well fitted to its own cluster and discriminated against other clusters). In this step, the objective is to by maximizing the silhouette coefficient (Rousseeuw 1987);

(2) Perform agglomerative clustering again using the absolute pattern $A_{i}^{TO_{k}}$ for every first-level type and generating *m*_2_ second-level types each. Here, *m*_2_ needs to be predefined in correspondence with the absolute volume level of human activities. For instance, if *m*_2_ = 3, there are three-level spatial units (i.e., low, medium, high) associated with human activity.

(3) Synthesize first-level and second-level types together to obtain the final *M*=*m*_1_×*m*_2_ human activity zones under *TO*_*k*_.

### Detecting spatio-temporal stratified heterogeneity (STSH)

In this study, we set the crime pattern as *Y* and human activity as *X*, respectively. Here, *M* strata (i.e., HAZs) is clustered from human activity (*X*) with *N* spatial units (*N*≥*M*). Then, Geodetector^2^ is used to determine the level of association (i.e., STSH) between HAZs and crime patterns across different TOs. Different temporal periods are used to examine the effect that the choice of time period has on the level of association. Specifically, the value of *Q* denotes the impact of human activity patterns on crime patterns in the study area during given observation time spans.

Spatial stratified heterogeneity (SSH) explores whether the observation within each stratum is significantly homogeneous but not between strata, i.e., within-strata variance is less than the between-strata variance. Accordingly, the method offered by *Geodetector* (Wang et al. 2010, 2016) is an efficient and novel way (without the restriction of statistical assumptions) to assess the SSH by testing the power of explanatory variable (independent) *X* (e.g., human activity) on a dependent *Y* (e.g., crime) by comparing homogeneity between the spatial distributions.

Geodetector assesses statistical association using a proposed *Q* statistic – *Q* value (the proportion of the between- to the within-strata variances) for measuring the heterogeneity. The greater the *Q* value is, the higher the heterogeneity at spatial units is:

1 $$ Q=1-\frac{1}{N \sigma^{2}} \sum_{h=1}^{M} N_{h}\sigma_{h}^{2},  $$

where *N* and *σ*^2^ refer to the number of spatial units and the variance of *Y* in a region, respectively. In addition, the explanatory *X* incorporates *M* strata (*h*=1,2,...,*M*)(*N*≥*M*). Specifically, *N*_*h*_ and $\sigma _{h}^{2}$ are the spatial unit numbers and the variance of *Y* in one stratum *h*, respectively. Here, the *M* strata can be determined by the explanatory *X* or *Y* itself. The larger the value of *Q*, the greater is the association between *X* and *Y*.

## Case study

### Data description and study area

Within the context of the global COVID-19 pandemic, the propagation of the virus in cities of the United States has rendered it as the country with the highest number of confirmed cases. San Francisco on the west coast of California, has been no exception and has seen a large number of recorded cases. In response to the pandemic, a SAH order, issued by the state government, was brought into force on Mar. 19, 2020, closely followed by a series of mandatory measures, such as the closure of non-essential business. Initial evidence of crime reduction in San Francisco during the SAH order at the city-level has been described in a recent empirical analysis (Ashby 2020).

In the current study, we use the central urban area consisting of 579 CBGs as the study area, as this area contains a wide variation of land uses and activity patterns. Data on reported crime incidents in San Francisco were obtained from the open data haven hosted by the San Francisco government^3^. This dataset compromises spatial and temporal information (i.e., the date, time and coordinates), neighbourhood information and the category of crime for each incident. The data used in this study covers two main crime types with 8 subcategories of crime. The property crime category consists of larceny theft, vandalism, motor vehicle theft, residential burglary and non-residential burglary; and the violent crime category consists of assault, domestic violence and robbery.

Human activities were represented in terms of direct footfall levels. This data was derived from more than 45 million anonymous mobile phone users and was processed and provided by SafeGraph^4^. The original footfall records were aggregated at the level of census block group (CBG) and at an hourly temporal scale. SafeGraph data provide unique and valuable insights into the foot-traffic changes in different areas of cities across the United States^5^. Here, we adopted the metric ”Stops by Day” in the database, which describes the number of device stops in an area each day (local time) to characterize the temporal human activity patterns at the CBG-level. The temporal time span of the data is from Feb. 7, 2020 to Apr. 30, 2020, i.e., six weeks before and after the commencement of the SAH order (Mar. 19, 2020).

### Human activity zones in different temporal observation periods

To enable the spatio-temporal detection of stratified heterogeneity, we manually defined six different TOs centred around Mar. 19, 2020, the day when stay-at-home (SAH) mandate started to take effect in San Francisco. The six periods are *TO*_1_: Mar. 13 to Mar. 26 (two weeks observation), *TO*_2_: Mar. 6 to Apr. 2 (four weeks observation), *TO*_3_: Feb. 28 to Apr. 9 (six weeks observation), *TO*_4_: Feb. 21 to Apr. 16 (eight weeks observation), *TO*_5_: Feb. 14 to Apr. 23 (ten weeks observation) and *TO*_6_: Feb. 7 to Apr. 30 (twelve weeks observation).

**Fig. 2:**
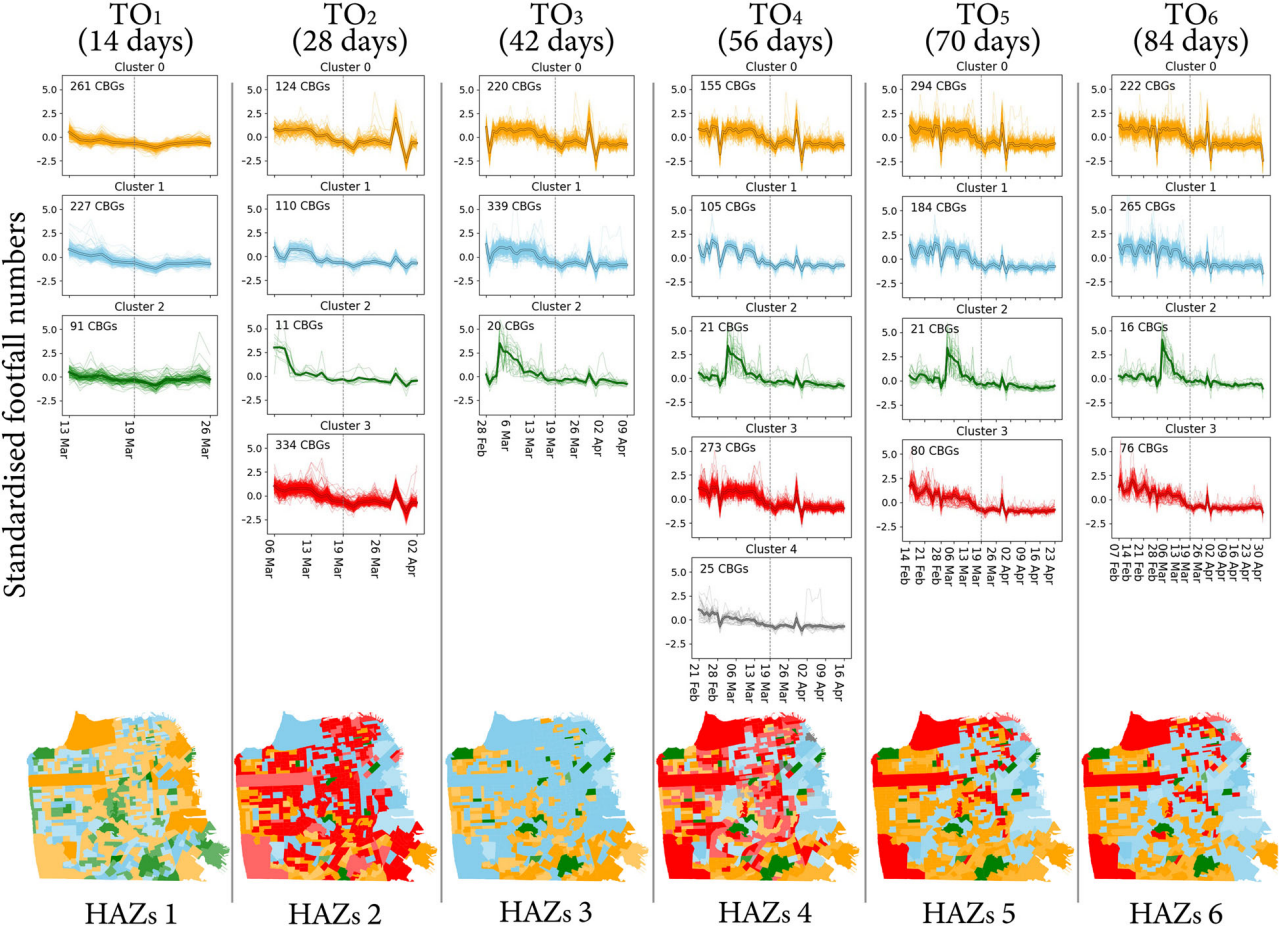
Clustering of temporal footfall patterns (top) and the corresponding human activity zones (bottom). The first-level clusters of temporal footfall patterns are illustrated as colored signatures for each TO. The bold center line for each signature cluster denotes its variation characteristic of human activities; Second-level clusters are distinguished by the color saturation in each map of human activity zones. Then, the HAZs in each TO are generated by the combination of first-level characterized by human activity pattern and second-level characterized by human activity volume with total zone numbers (m1 x m2), i.e., 9 (3×3), 12 (4×3), 9 (3×3), 15 (5×3), 12 (4×3), 12 (4×3)

For each TO, we performed the functional zoning outlined in Section 2.2 to generate the HAZs respectively. The clustered temporal footfall patterns and the corresponding HAZs for the six TOs are illustrated in Fig. 2. The number of first-level types (*m*_1_) was iterated from 2 to 10 and determined based on the elbow plot of silhouette coefficients (Rousseeuw 1987). As a result, we found the optimum numbers for *m*_1_ for *TO*_1_,*TO*_2_,*TO*_3_,*TO*_4_,*TO*_5_,*TO*_6_ to be 3, 4, 3, 5, 4, 4 respectively. These first-level types are labelled as cluster 0 (in yellow) up to cluster 4 (in grey) in Fig. 2. CBGs with similar footfall patterns are labelled as the same cluster and then grouped into a HAZ. We then assume *m*_2_=3 to further discriminate three second-level types for each first-level cluster (Zhu et al. 2017) according to the absolute footfall numbers. This allows the classification of variation in types within the first level clusters. After the second-level classification, we could have *m*_1_×*m*_2_ total clusters for each time period.

For interpretation, we apply different color hues to denote the first-level clusters and use saturation of the same color to denote the second-level clusters under the same first-level type. Thus, we obtained six different spatial regionalization of HAZs for the six TOs. On the bottom of Fig. 2, the CBGs are visualized according to their types. Noting that CBGs of the same clustered label are plotted with the same color hue and saturation, these CBGs are aggregated into HAZs in the following detection of stratified associations (Section 3.4). More specifically, the number of generated HAZs for the six TOs are 9, 12, 9, 15, 12, 12, respectively.

It is interesting to observe that the spatial distributions of human activity patterns vary substantially across TOs. Overall, we can retrieve several temporal clusters and HAZs from human activity patterns for each TO. However, the clustering results of human activity patterns observed under *TO*_1_ are not as discriminative and informative as other time spans (i.e., temporal patterns tend to have similar steady-state), while *TO*_4_ produces the most heterogeneous regionalization both temporally and spatially, with many local variations of HAZ types. These dynamic HAZ classification imply a complex set of typologies in terms of the changes in human activity patterns across the SAH restrictions period. For example, under *TO*_4_, cluster 2 in green shows a spike in activity between Feb.28 and Mar.6 before the SAH order which is not seen in other clusters. In addition, though the change can be observed between Mar.26 and Apr.2 after SAH order in every cluster in *TO*_4_, cluster 0 denotes the highest degree of magnitude of such change (from standardized values 4 to -4).

### Crime spatial distributions on CBGs

To correspond with the human activity pattern observation timespans, the crime pattern in our study are represented by the distribution of crime numbers at the CBG-level across the six defined time periods. To illustrate, here we use the example of the sixth time observation (*TO*_6_) (Feb. 7 to Apr. 30). Over this time period, there were a total of 14311 crime incidents consisting of 11460 property crimes (including 2538 larceny thefts; 1855 vandalism, 1418 motor vehicle thefts, 451 residential burglaries and 1198 non-residential burglaries) and 2851 violent crimes (including 1599 assaults, 572 domestic violence and 680 robberies).

**Fig. 3:**
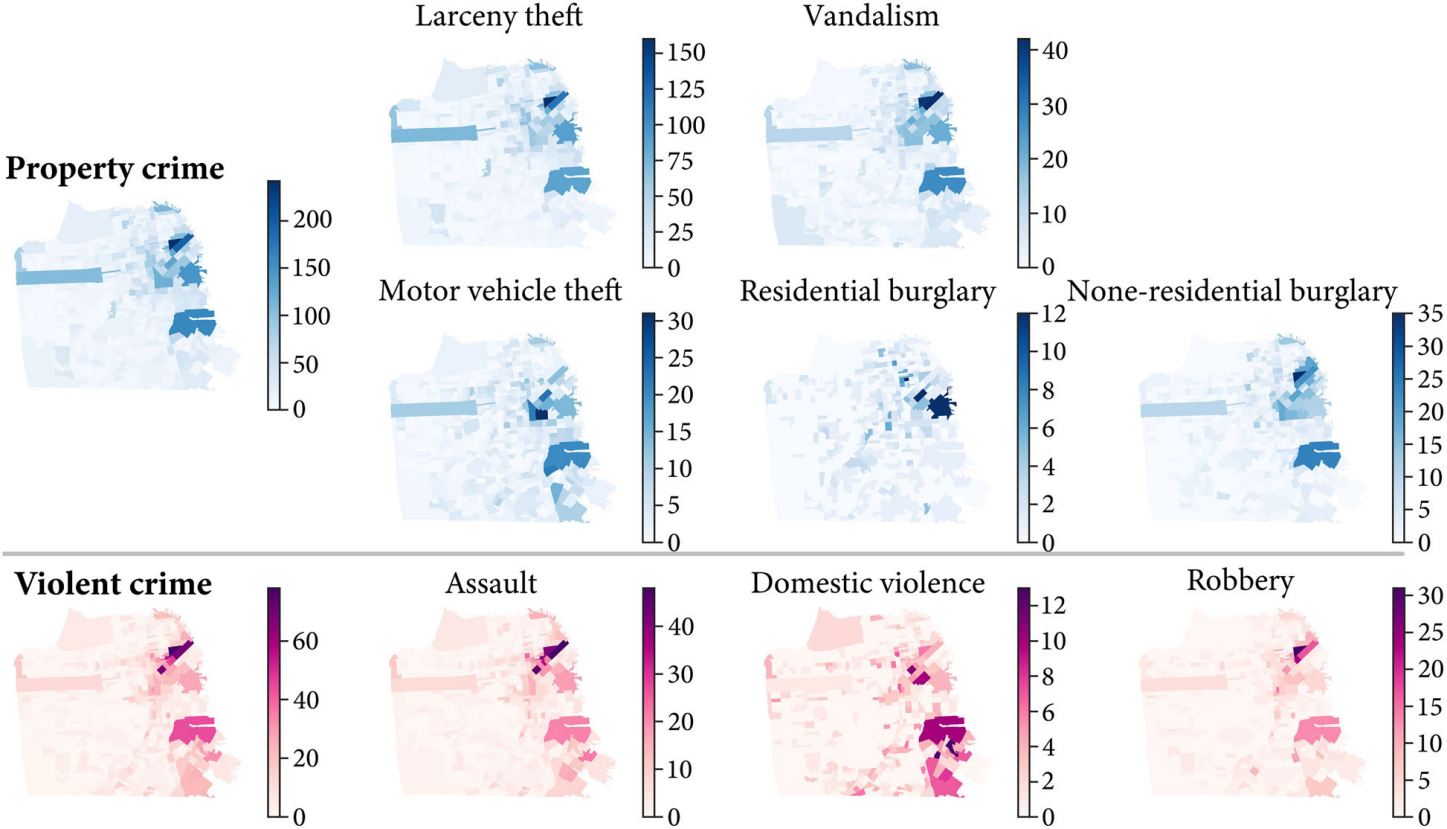
Spatial distribution of crime in different types at CBG-level during the sixth observation timespan (*TO*_6_) (Feb. 7 to Apr. 30)

The spatial distribution of these types of crime and their concentration within CBGs are visualized in Fig. 3. The two main types of crime are denoted by two colors; property crimes are represented in blue and violent crimes are represented in purple. In each crime map, the level of transparency denoted the volumes of crime incidents occurring in the CBGs. As expected given the evidence on the law of crime concentration at place (Weisburd 2015) there is significant clustering of crime. Generally, the crime of all types are mainly distributed on the east side of San Francisco where residential blocks and commercial areas are located. Although there are differences in the overall numbers of property crime and violent crime, their distributions show similar patterns, demonstrating that crime concentrates on several distinctive CBGs. Furthermore, residential burglary appears clustered on limited CBGs and has a distinct individual pattern compared to other property crimes. Similarly, domestic violence is distinctively clustered on the southeast side of the study area suggesting that this type of crime is strongly related to the specific urban zone features. In summary, there is evidence that the crime pattern strata are distinctive across the eight subtypes of crime and one explanation for this heterogeneity lies in the differences of human activity patterns as measured by the HAZs.

### Spatio-temporal stratified associations

The spatio-temporal stratified association between human activities and crime patterns across six TOs and eight subtypes of crime are reported in Table 1. As expected, the crime pattern strata are strongly associated with the distribution of HAZs. In most cases, there is a positive and significant association between crime and HAZ categorizations. So, different levels of crime can be well discriminated using area types with distinct activity patterns. Hence, human activity patterns are a good explanatory variable in addressing the spatial heterogeneity of urban crime. Indeed, the amplified differences in HAZ patterns caused by the COVID restrictions appear to have given a strong test of the association between routine activities and property crime (Halford et al. 2020).

**Table 1:** The results of STSH index (Q statistics) between crime distribution and HAZs across six TOs in 2020

	2 weeks	4 weeks	6 weeks	8 weeks	10 weeks	12 weeks
**All crime**	0.292***	0.418***	0.292***	0.446***	0.408***	0.414**
**Property crime**	0.291***	0.420***	0.298***	0.455***	0.430***	0.435***
Larceny theft	0.227***	0.404***	0.290***	0.430***	0.420***	0.426***
Vandalism	0.209***	0.300***	0.293***	0.397***	0.370***	0.362***
Motor vehicle theft	0.086***	0.102**	0.057	0.184***	0.182***	0.194***
Residential burglary	0.018	0.024	0.042	0.060	0.064*	0.123**
None-residential burglary	0.165***	0.305***	0.226***	0.416***	0.391***	0.399***
**Violent crime**	0.125***	0.223***	0.161***	0.288***	0.225***	0.225***
Assault	0.085***	0.204***	0.142***	0.251***	0.201***	0.214***
Robbery	0.111***	0.159***	0.150***	0.253***	0.221***	0.233***
Domestic violence	0.036*	0.056*	0.050*	0.111***	0.061*	0.047

* p<0.05, **p<0.01, ***p<0.001

In terms of the two main crime types, the property crime displays greater association indices (0.446 as the highest value) than violent crime (0.288 as the highest value) across all six *TOs*. The fact that property crime patterns are strongly impacted by the human activity zones is likely related to the underlying crime generation mechanism. Many property crimes are strongly related to the opportunities which occur when motivated offenders and potential victims/targets converge in space and time. Conversely, violent crime is more weakly associated with human activity patterns, indicating that violent crime might be (in part at least) driven by mechanisms other than the crime opportunities due to routine activity dynamics. Statistically, the weaker associations could also be related to the more limited reporting of violent incidents to the police which results in a lower variance across HAZs according to STSH.

Mixed findings emerged when further exploring the associations between crime and human activity across the subtypes of crime. In terms of property crime, larceny theft and non-residential burglary are most strongly connected with human mobility patterns. This could potentially be due to SAH order causing restrictions in access to the places where and the targets against which these types of crime mostly occur. Interestingly, residential burglary does not appear as strongly tied to the HAZ classifications. This might be related to the nature of the data used in categorization – device stops are likely to differentiate more strongly between activities away from the home. In parallel, for violent crime, the finding that the pattern of assault and robbery are more influenced by human activity patterns than domestic violence appears intuitive, as again these rely on available opportunities and targets away from the home.

Concerning the temporal heterogeneity in the association between crime and human activity, all crime types exhibit the highest *Q* values (0.430 in larceny theft as the largest value) in *TO*_4_ (8 weeks of observation timespans) compared to the other *TOs*. It is intriguing that crime patterns are most strongly impacted by HAZs based on the similarity of human activity patterns before and after 4 weeks around the SAH order. A potential explanation for this is that patterns in different crime types influenced by human activity change in the pandemic have a preferred diffusion time in terms of their effect. For example, residential burglary has no statistically significant association with human activity in the short-term TOs until these span 10 weeks, which might suggest that only the comparably longer-term patterns in residential burglary in the pandemic can be explained by activity changes. It is also evident that the strongest associations are shown for the time period over which the clustering shows the most discrimination between HAZ categories (i.e., *TO*_4_ in Fig. 2.)

**Fig. 4:**
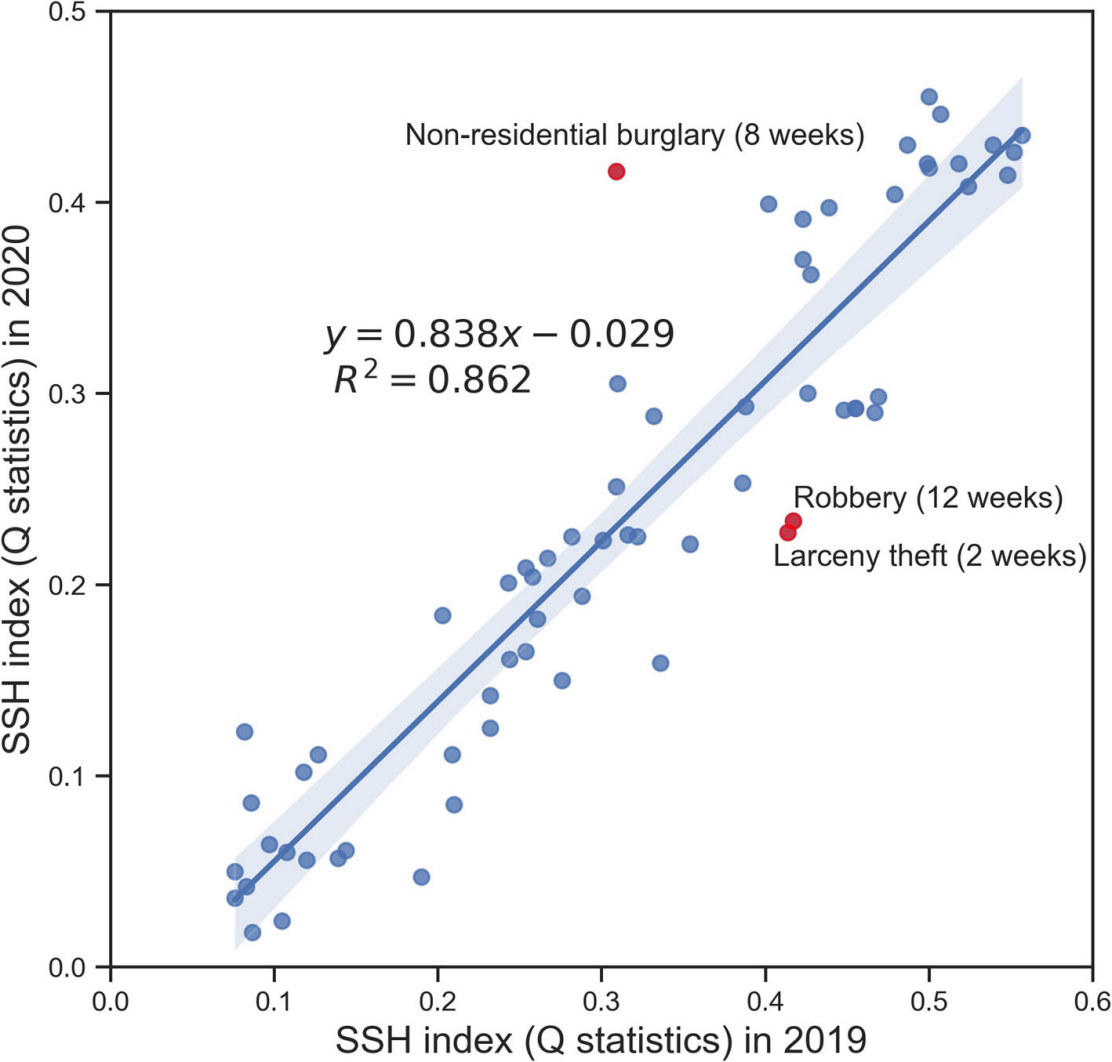
Linear regression of STSH index (Q statistics) between 2019 and 2020 (The shadow area represents confident interval of 0.99)

For a comparison with non-pandemic times, we used the same data sources for 2019 to generate STSH indices for the same selected crime types following the same analytic framework. For a fair comparison, we set six TOs (2 weeks, 4 weeks, 6 weeks, 8 weeks, 10 weeks and 12 weeks) around Mar. 19, 2019. For each TO, the STSH index was calculated to represent associations between the spatial distribution of crime (2 types with 8 subtypes) and the human activity pattern represented by HAZs regenerated for 2019. The association indices are shown in Table 2. In addition, Fig. 4 shows a linear regression (LR) of STSH indices between 2019 and 2020, which denotes the consistent degree of impact strength of human activity on the crime patterns across these two years.

**Table 2:** The results of STSH index (Q statistics) between crime distribution and HAZs across six TOs in 2019

	2 weeks	4 weeks	6 weeks	8 weeks	10 weeks	12 weeks
**All crime**	0.455***	0.500***	0.455***	0.507***	0.524***	0.548***
**Property crime**	0.448***	0.499***	0.469***	0.500***	0.539***	0.557***
Larceny theft	0.414***	0.479***	0.467***	0.487***	0.518***	0.552***
Vandalism	0.254***	0.426***	0.388***	0.439***	0.423***	0.428***
Motor vehicle theft	0.086	0.118***	0.139***	0.203***	0.261***	0.288***
Residential burglary	0.087	0.105**	0.083	0.108	0.097	0.082
None-residential burglary	0.254***	0.310***	0.316***	0.309***	0.423***	0.402***
**Violent crime**	0.232***	0.301***	0.244***	0.332***	0.282***	0.322***
Assault	0.210***	0.258***	0.232***	0.309***	0.243***	0.267***
Robbery	0.209***	0.336***	0.276***	0.386***	0.354***	0.417***
Domestic violence	0.076*	0.120***	0.076	0.127***	0.144***	0.190***

* p<0.05, **p<0.01, ***p<0.001

In general, the impact of human activity on crime pattern represented by the STSH index values in 2020 are lower than those in the non-pandemic year of 2019 (*coefficient*<1,*constant*<0). There are also differences in the size of the STSH statistics between various crime types within TOs for 2019. Comparing the STSH indices across the years demonstrates that, the only statistically significant increase in the STSH index between the years is for non-residential burglary (TO is 8 weeks); the largest declines in the STSH index are for robbery (TO is 12 weeks) and larceny theft (TO is 2 weeks). These outlier points are plotted as red color in the Fig. 4. The differences in strength of association between the year before and the year within the pandemic are interesting. The generally weaker associations in the pandemic period might indicate a degree of offender adaptation (Borrion et al. 2020; Gradoń 2020) – where target seeking is slightly less associated with opportunities provided by human activity patterns. However, the fairly linear relationship across the years appears to indicate that any adaptation does not appear to be exclusive to particular crime types. A further explanation is that the activity pattern differences across the HAZ profiles were less pronounced (and therefore discriminatory) for the pandemic period as movement restriction means there were likely to be less dramatic spatial differences in populations on streets during the lockdown period.

## Discussion

This study aimed to investigate the spatial-temporal stratified association between human activities and crime patterns within the dynamic urban context. Our analytic framework has demonstrated that spatial-temporal stratified association as measured by the STSH index can be an effective way of exploring the relationship between human activity patterns and spatial crime patterns in CBGs in San Francisco city. In our framework, first, the human activity patterns are captured by identifying HAZs using hierarchical clustering algorithms. Then, the STSH is examined as a measure of association between human activity stratified patterns at HAZ-level and spatial crime strata patterns at CBG-level across different observed time periods.

Overall, the findings that human activity has significantly impacted crime across different HAZs and observed temporal periods during the COVID-19 pandemic are in line with expectations under crime pattern theory. However, the human activity pattern that influences on property crime appears stronger than violent crime. This suggests that crime opportunity generation for property crime is more closely coupled to the activity dynamic of humans in urban environments. Another layer of explanation uses the concept of crime generators (Brantingham and Brantingham 2008; Brantingham et al. 2017); those locations most frequently visited by people, e.g., supermarkets, public transportation, are likely to generate large amounts of property crime. The discrepancies in the influence of human activity patterns between property crime and violence suggest the role of different complex mixes of criminogenic factors and drivers for different crime types. Most property crime (except residential burglary) occurs in the urban areas with high footfall, whilst violent crimes (e.g., domestic violence) are more weakly connected with the human activity variation in the urban areas.

Considering the COVID19 pandemic consequence of human activity reduction due to SAH orders, most associations between human activity patterns and crime patterns, as measured by the STSH index, show statistically significant results either in 2019 or 2020 around the predefined observation date or in both years. However, we found that the level of association between human activity and crime in 2019 (pre-pandemic) is generally higher than in 2020. In line with the routine activity theory, the three main factors influencing crime opportunity (motivated offenders, available potential victim/targets and lack of guardianship) are unlikely to have been affected equally across crime types in the context of the human activity decreases caused by the SAH order in 2020. For example, in a reversal of the general trends, the association between human activity patterns and non-residential burglary (TO is 8 weeks) in 2020 was higher than it was in 2019. Obviously, the impact of human activity reduction will be emphasized for this crime due to the shutdown of commercial sites, supermarkets, bars and restaurants and might reflect the reduction in guardianship.

It is worth emphasizing that this study has shown relationships between *types* of human activity pattern (in terms of temporal profile and volume) and crime *types* do not support a consistent general rule between decreases in activity and subsequent decreases in crime. It has shown that these varying relationships exist regardless of large-scale mobility changes such as those seen in the pandemic. Dis-aggregating some of the results presented here could identify the types of HAZs that are significantly associated with higher or lower levels of distinct crime types. Doing this might provide evidence that assists with developing tailored intervention recommendations. For instance, policing resources might be more responsive and more efficiently used if additional policing patrolling was allocated in a dynamic way to areas that exhibit strong and significant associations between human activity and crime.

## Conclusions

In conclusion, this research analyzed human activities and crime patterns at the CBG-level and explored the spatio-temporal stratified associations between the two variables in San Francisco around the COVID-19 SAH mandate. The results facilitate our understanding of how human activity patterns could be impacted by COVID-19 containment policies, and thus affect crime patterns differently across geographic space and crime types. The examination of spatio-temporal stratified association can be adopted as an approach to disentangle some of the complex relationships between activity and crime. The findings not only strengthen our empirical knowledge concerning the relationships between urban crime and human activities, but also give insight that crime intervention strategies could be developed using consideration of human activity related variables. Moreover, the fact that crime can be explained by human activity data sheds light on the promising future of combining crime science with a wide range of geospatial data analysis.

Following the proposed analytical framework, HAZs can be efficiently generated based upon the CBGs using agglomerative clustering algorithms, and the spatio-temporal stratified association matrix can then be calculated across crime types and observation time periods using Geodetector method. In our clustering part of this framework, reducing the uncertainty of HAZ numbers needs to be concerned as the HAZ generation optimization is determined by the hierarchy clustering methods, especially the manually predefined parameter in the second level clustering. One limitation regarding the use of STSH to measure the association between human activity dynamics and crime patterns is that the method used here neglects the impact of seasonality. In this initial exploration, it has not been possible to examine differences in patterns in mobility patterns in time of the day or day of the week. Another concerning issue is that we only compare the associations (between human activity and crime) in 2019 and 2020 to emphasis the lockdown influence under the same set of static explanatory variables. However, the human activity patterns could be the reflection of other dynamic underlying driving factors. Dynamic human activity involving other controlling variables need to be examined in the further work. Additionally, the internal spatial heterogeneity of HAZs which also affect the crime patterns needs to be more closely considered in future research as a lack of attention to this might impede optimum classification.

## Data Availability

Data and materials will be published at https://github.com/tongxinchen/Crime_HAZ_SF_USonce the paper is accepted.

## Footnotes

1 https://scikit-learn.org/stable/modules/clustering.html#hierarchical-clustering

2 https://cran.r-project.org/web/packages/geodetector/index.html

3 https://data.sfgov.org/Public-Safety/Police-Department-Incident-Reports-2018-to-Present/wg3w-h783

4 SafeGraph (https://www.safegraph.com/) is a data company that offers location data, spatial hierarchy metadata and place traffic data aggregated from numerous applications to provide insights about the physical world.

5 Data schema available at https://docs.safegraph.com/docs/neighborhood-patterns

## Code availability

Code to reproduce the work will be available at https://github.com/tongxinchen/Crime_HAZ_SF_US once the paper is accepted.
